# Psychotic experiences and disorders in adolescents and young adults with borderline intellectual functioning and intellectual disabilities: evidence from a population-based birth cohort in the United Kingdom

**DOI:** 10.1017/S0033291724003556

**Published:** 2025-02-05

**Authors:** Christina Dardani, Jack Underwood, Hannah Jones, Alexandros Rammos, Sarah Sullivan, Laura Hull, Golam Khandaker, Stan Zammit, Dheeraj Rai, Paul Madley-Dowd

**Affiliations:** 1 MRC Integrative Epidemiology Unit, Bristol Medical School, University of Bristol, Bristol, UK; 2 Population Health Sciences, Bristol Medical School, University of Bristol, Bristol, UK; 3 Lovisenberg Diaconal Hospital, Oslo, Norway; 4 PsychGen Centre for Genetic Epidemiology and Mental Health, Norwegian Institute of Public Health, Oslo, Norway; 5 Neuroscience and Mental Health Innovation Institute, School of Medicine, Cardiff University, Cardiff, UK; 6 Centre for Neuropsychiatric Genetics and Genomics, School of Medicine, Cardiff University, Cardiff, UK; 7 National Institute for Health Research Biomedical Research Centre, University of Bristol, Bristol, UK; 8 Department of Clinical, Educational & Health Psychology, University College London, London, UK; 9 Bristol Autism Spectrum Service, Avon and Wiltshire Partnership NHS Mental Health Trust, Bristol, UK

**Keywords:** ALSPAC, intellectual disabilities, psychosis, trauma

## Abstract

**Background:**

Individuals with borderline intellectual functioning and intellectual disabilities (intellectual impairment) may be at increased risk of psychosis. However, studies have been limited by small and selected samples. Moreover, the role of early life trauma, a key risk factor for psychosis, in the associations is unknown.

**Methods:**

Using data from the Avon Longitudinal Study of Parents and Children (ALSPAC) birth cohort, we investigated the associations between intellectual impairment, psychotic disorders, and psychotic experiences, and assessed the mediating role of trauma in childhood. Individuals with intellectual impairment were identified using a multisource measure utilizing indicators from ALSPAC combined with health and administrative records. Psychotic disorder diagnoses were extracted through linkage to primary care records. Psychotic experiences were assessed at ages 18 and 24 using the semi-structured Psychosis-Like Symptoms interview (PLIKSi). Trauma between ages 5 and 11 was assessed with questionnaires and interviews administered to children and parents at multiple ages. Multiple imputation was performed to mitigate bias due to missing data.

**Results:**

The maximum sample after multiple imputation was 9,407. We found associations between intellectual impairment and psychotic disorders (OR = 4.57; 95%CI: 1.56–13.39). Evidence was weaker in the case of psychotic experiences (OR = 1.63; 95%CI: 0.93–2.84). There was some evidence suggesting a mediating role of trauma in the associations between intellectual impairment and psychotic experiences (OR = 1.09; 95%CI: 1.03–1.15). Complete records analyses yielded comparable estimates.

**Conclusions:**

Intellectual impairment is associated with psychotic disorders and experiences in adulthood. Research into the contribution of trauma could shape intervention strategies for psychotic disorders in this population.

## Introduction

Borderline intellectual functioning and Intellectual disabilities (henceforth intellectual impairment)refer to difficulties in cognitive and adaptive functioning that manifest early in childhood and have a substantial impact on education, independent living skills, employment, and access to social support and health care across the lifespan (Patel et al., 2018). Intellectual impairment has a neurodevelopmental origin and is not the result of later neurocognitive changes due to injury or disease. Recent meta-analytic evidence suggests that the lifetime prevalence of mental health conditions in intellectual impairment may be higher than the general population (pooled lifetime prevalence: ≈32% vs ≈ 29% in the general population)(Mazza et al., 2020; Steel et al., 2014). Co-occurring mental health conditions in intellectual impairment have been associated with adverse behavioral, educational, and social outcomes for the affected individuals as well as lower quality of life for their families and carers (Maes et al., 2003). On this basis, understanding the links between intellectual impairment and mental health conditions is among the top global research priorities in the field (Tomlinson et al., 2014).

Affective and non-affective psychotic disorders (henceforth psychotic disorders) are among the most common co-occurring mental health conditions in intellectual impairment. The prevalence of psychotic disorders in intellectual impairment appears to be higher than their estimated lifetime prevalence in the general population, with schizophrenia reaching approximately 4.8% (vs ≈ 0.9% in the general population), unspecified psychotic disorder reaching 3.9% (vs ≈ 0.5%) and bipolar disorder approximately 2% (vs ≈ 0.2%)(Aman et al., 2016; Buckley et al., 2020; Mazza et al., 2020; Perälä et al., 2007). However, most studies so far have been limited by small and selected samples (predominantly inpatient), with limited control for potential confounding factors. Furthermore, these studies have been predominantly based on diagnoses of psychotic disorders in this population (Mazza et al., 2020), offering limited insights into sub-clinical symptoms and potential precursors of psychotic disorders, such as psychotic experiences.

Several factors have been proposed to influence the risk of psychiatric conditions in individuals with intellectual impairment, including access to support services, poor physical health, and major life events (including but not limited to trauma)(Allan et al., 2007; Cooper et al., 2007). The latter is particularly relevant in the case of psychotic disorders. Traumatic life events (such as physical, sexual, and emotional abuse, neglect, domestic violence, and bullying victimization) are among the most consistently identified risk factors for psychotic experiences and disorders in the general population (Croft et al., 2019; Varese et al., 2012). Emerging evidence suggests high rates of exposure to traumatic life events in the population with intellectual impairment (Berg et al., 2019; Wigham & Emerson, 2015). There is an absence of studies investigating whether and to what extent traumatic life events mediate any associations between intellectual impairment and psychotic experiences and disorders.

Using prospectively collected questionnaire, interview, and health record linkage data in a population-based birth cohort in the UK, we assessed: (A) the risk of psychotic disorders and psychotic experiences during early adulthood in individuals with and without intellectual impairment, (B) the potential associations of intellectual impairment to longitudinal profiles of psychotic experiences reflecting the persistence and frequency of psychotic experiences from age 12 to age 24 (C) the extent to which trauma experienced in childhood may mediate the links between intellectual impairment and psychotic disorders and psychotic experiences, (D) the possible confounding influence of familial, socioeconomic and demographic factors in any identified links.

## Methods

### Study design and participants

**Figure 1:**
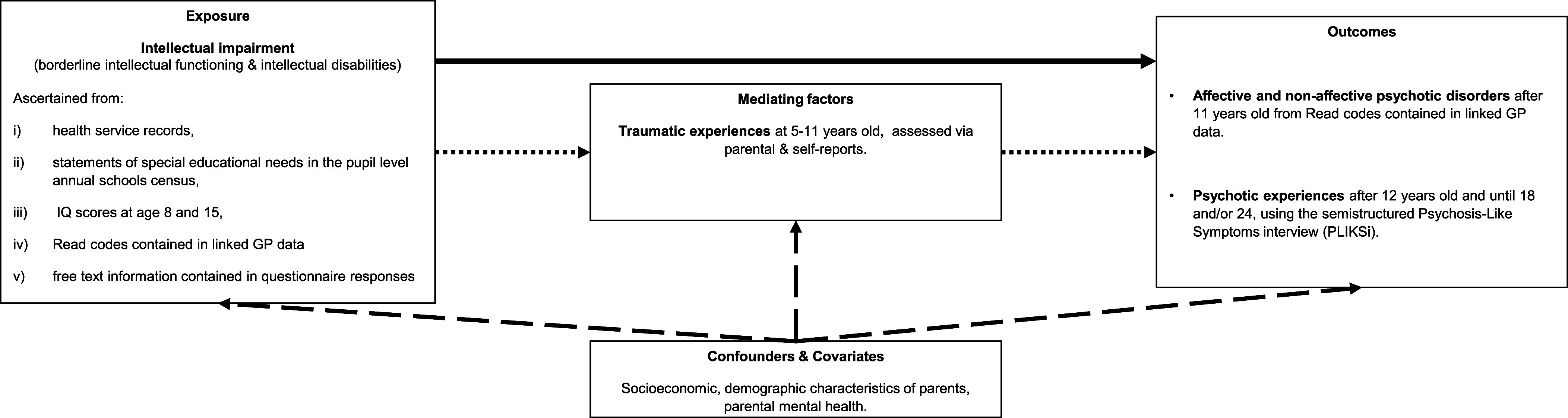
Visual summary of the study aims and design. Solid black lines correspond to the analyses investigating the links between intellectual impairment and psychotic disorders and experiences. Dotted black lines correspond to the analyses investigating the extent to which any links between intellectual impairment and psychotic disorders and experiences are mediated via traumatic experiences in childhood. Dashed black lines correspond to the analyses investigating the possible confounding influence of familial, socioeconomic, and demographic factors in the links between intellectual impairment and psychotic disorders and experiences. Please note that intellectual impairment in the context of the present study refers to borderline intellectual functioning and intellectual disabilities that are typically of neurodevelopmental origin and therefore in the present study they were assumed to precede the mediator and outcome.

A visual summary of the study’s aims and design can be found in Figure 1. The Avon Longitudinal Study of Parents and Children (ALSPAC) is a population-based cohort study of children born to 14,541 pregnant mothers residing in the former county of Avon, United Kingdom, with an expected delivery date between 1 April 1991 and 31 December 1992. Of these initial pregnancies, there was a total of 14,676 fetuses, resulting in 14,062 live births and 13,988 children who were alive at 1 year of age. When the oldest children were approximately 7 years of age, eligible participants who did not join the study initially were contacted, and additional participants were recruited. This resulted in a total of 15,447 pregnancies and 15,658 fetuses, of which 14,901 were alive at 1 year of age.

Further information on the ALSPAC cohort is available on the ALSPAC website (http://www.bristol.ac.uk/alspac) and elsewhere (Boyd et al., 2013; Fraser et al., 2013; Northstone et al., 2019). The study website contains details of all the data that is available through a fully searchable data dictionary and variable search tool (http://www.bristol.ac.uk/alspac/researchers/our-data/). Some study data were collected and managed using REDCap electronic data capture tools hosted at the University of Bristol. REDCap (Research Electronic Data Capture) is a secure, web-based software platform designed to support data capture for research studies. (Harris et al., 2009, 2019).

For data collected via questionnaires and clinics, informed consent was obtained from participants following the recommendations of the ALSPAC Ethics and Law Committee at the time. Ethical approval for the study was obtained from the ALSPAC Ethics and Law Committee and the Local Research Ethics Committees (NHS Haydock REC: 10/H1010/70).

#### Linkage to health and administrative records

When the index children reached legal adulthood (age 18), ALSPAC conducted a postal fair-processing campaign to re-enroll them into the study and to seek permission for linkage to health and administrative records. This was an ‘opt-out’ approach, meaning linkage was attempted for all participants, except those who objected and those who were not sent fair processing materials. Where ‘opt-in’ consent became practicable (e.g., when a participant attended a study assessment visit) then this was collected by a trained fieldworker. Details on the linkage process can be found elsewhere (Cornish et al., 2021).

In the context of the present study, the eligible sample was defined based on the following criteria: (1). having available linkage data and not dissenting to their use, (2). having linked primary care data available from age 11 onwards (considering that we were interested in the adolescent and early adulthood period). A total of 9,680 participants were eligible.

### Measures

#### Exposure: intellectual impairment

Intellectual impairment was identified in ALSPAC using a composite measure created in previously published work (Madley-Dowd et al., 2022) based on information from six different sources: (i) IQ scores less than 85 measured at age 8 and age 15, (ii) free text fields from parent-reported questionnaires, (iii) school-reported provision of educational services for individuals with a statement of special educational needs for cognitive impairments, (iv) from relevant Read codes (Chisholm, 1990) contained in GP records (Read codes are a comprehensive list of standardized clinical terms used by healthcare professionals within the UK National Health Service to record clinical information), (v) international classification of disease (ICD) diagnoses contained in hospital episode statistics and (vi) recorded interactions with mental health services for ID contained within the mental health services data set. The intellectual impairment measure is available on the UK Secure eResearch Platform (UKSeRP). Further details on the creation of the composite intellectual impairment measure can be found in the original publication (Madley-Dowd et al., 2022).

Considering that our exposure definition captured a broad group of individuals with intellectual impairment (IQ < 85), we additionally used a stricter definition of intellectual disabilities. Specifically, intellectual disabilities were defined using the same measures and criteria described above, with the only difference in criteria being an IQ score less than 70, where IQ information was available.

#### Primary outcome: psychotic disorders

Read codes (V.2) from GP records relevant to the diagnosis and symptoms of psychotic disorders were extracted to identify the outcomes of interest in the eligible sample. GP records were available from 1990 to 2016 when the oldest participants were 25 years of age. The full list of reading codes used can be found in the project-dedicated repository: https://github.com/pmadleydowd/BailyThomas-IntellectualDisability-and-MentalHealth.

#### Primary outcome: psychotic experiences until early adulthood

Psychotic experiences were assessed at ages 18 and 24 using the semi-semi-structured Psychosis-Like Symptoms interview (PLIKSi), administered by trained psychologists, and scored according to criteria predefined by the World Health Organization (Organization, 1994). The PLIKSi consists of 12 core questions covering hallucinations, delusions, and thought interference. Participants were asked about experiences that had occurred since age 12 years. Psychotic experiences were considered present if, at ages 18 and/or 24 years, one or more of the experiences was rated by the interviewer as suspected or definitely present, and if this was not attributable to falling asleep or waking up or fever. We additionally examined psychotic experiences that had been distressing and/or frequent, since these experiences are more clinically relevant and predictive of psychotic disorder (Sullivan et al., 2020).

#### Secondary outcome: longitudinal profiles of psychotic experiences

Considering that psychotic experiences are, in most cases, transient in the general population (Sullivan et al., 2020), and do not necessarily reflect liability to psychotic disorders later in life, we additionally used a measure reflecting the persistence and frequency of psychotic experiences across three time points in ALSPAC: ages 12, 18, and 24 years. Details on the measure can be found in the original publication (Rammos et al., 2021). Briefly, using information from the PLIKSi on current presence (over the past 6 months) and frequency of psychotic experiences (0: “Not present,” 1: “Low-frequency” – experiences occurring less than weekly, 2: “High-frequency” – experiences occurring weekly or daily), at each time point, an empirical composite measure was generated reflecting four longitudinal profiles of psychotic experiences from ages 12–24: (A). No experiences: Individuals without a psychotic experience at any time point; (B). Transient: Individuals with a psychotic experience rated at only one time-point, regardless of frequency; (C). Low-frequency persistent: Individuals with low-frequency psychotic experiences at two or more time points, or with a low-frequency rating at one time point and a high-frequency rating at another; (D). High-frequency persistent: Individuals with high-frequency psychotic experiences rated at two or more time points. Following previous work we did not make an assumption on the potential severity ordering of the profiles (Rammos et al., 2021), particularly considering that the boundaries between the transient and persistent-low profiles might be difficult to define.

#### Mediator: traumatic experiences in childhood

The measures of childhood trauma and their associations with psychotic experiences have been described in detail elsewhere (Croft et al., 2019). In brief, we used a measure of childhood trauma between ages 5 and 11 based on responses to 57 questions from questionnaires and interviews about domestic violence (regular acts of physical violence taking place in the home), physical abuse (physical harm to the participant from caregivers or other adults), emotional abuse (emotional cruelty to the participant from caregivers or other adults), emotional neglect (caregivers not taking an interest in the participant’s life), sexual abuse (adults or older children forcing the participant into sexual activity, including attempts to do so), and bullying victimization (regular name-calling, blackmail, or assault by peers). Measures of sexual, physical, and emotional abuse, assessed contemporaneously by the participant and their caregivers between participant ages 5 to 11, were supplemented with data from a participant-completed questionnaire at age 22, as all data on sexual abuse, and most data on physical and emotional abuse prior to age 11, were based on parental report. Each type of trauma was coded as present or not, and a single trauma variable was created representing exposure to any type of trauma (Croft et al., 2019).

#### Covariates

Covariates in the present study were selected on the basis of their potential associations with the exposure, outcomes, and mediator. These included child sex (male/female), maternal parity (≤1 child versus ≥2 children), major financial problems in the family when the child was 8 months old (yes/no), maternal highest educational attainment (32 weeks gestation), maternal age (at delivery), maternal Crown-Crisp anxiety scores (Crown & Crisp, 1966) (18 weeks gestation), and maternal depression measured with the Edinburgh Postnatal Depression Scale (Cox et al., 1987) (EPDS; 18 weeks gestation scores ≥13). Moreover, in order to better capture socioeconomic position which has been consistently associated with intellectual impairment, we added in our association analysis models as covariates: car ownership status (owning/not owning, 8 weeks of gestation), maternal marital status (married/separated, 8 weeks of gestation) and home ownership status (owned/rented, 8 weeks of gestation). This decision was based not only on the information they could provide but also on their completeness in the eligible sample (ranging from 87–88%).

### Statistical analyses

#### Association analyses

Statistical analyses were conducted in StataSE version 18. We estimated descriptive statistics of participant characteristics for individuals with and without intellectual impairment, traumatic experiences, and psychotic experiences.

Using logistic regression, we estimated odds ratios (OR) and 95% confidence intervals (95% CI) for the associations between intellectual impairment and psychotic disorders as well as psychotic experiences in early adulthood. Using g-computation via Stata’s margins command, we further estimated the adjusted marginal risk (overall covariates) and risk difference of each outcome for participants with and without ID. Standard errors were calculated using the delta method. Using multinomial logistic regression, we estimated relative risk ratios (RRRs) and 95% CIs for the associations between intellectual impairment and the four longitudinal profiles of psychotic experiences. Across all association analyses, we performed crude and covariate-adjusted models.

We additionally conducted association analyses using intellectual disabilities (including IQ < 70) as the exposure. It is worth noting that these analyses were expected to have substantially lower power than the ones using intellectual impairment as the exposure, but nevertheless informative on the potential associations between intellectual disabilities, psychotic disorders, and experiences.

#### Mediation analyses

We decided to conduct mediation analyses between intellectual impairment (exposure), trauma (mediator), and psychosis-related outcomes regardless of whether there was evidence of associations between intellectual impairment and the outcomes of interest. This decision was based on previous work suggesting that evidence of associations between exposure and outcome should not guide decisions for subsequent mediation analyses, particularly when the effect size is expected to be small or there may be suppression effects (when the direct and indirect effects of an exposure on an outcome have opposite directions)(O’Rourke & MacKinnon, 2018; Shrout & Bolger, 2002). Mediation analyses were performed using the g-formula package (Daniel et al., 2011) in Stata. We used the parametric g-formula using 10,000 Monte Carlo simulations to estimate the natural direct effect (NDE) of intellectual impairment on psychotic experiences, and the natural indirect effect (NIE) that was mediated via traumatic experiences between ages 5 and 11. We performed crude as well as covariate-adjusted models. Corresponding 95% CIs were estimated using the standard errors from 1000 nonparametric bootstrap resamples.

#### Missing data

Considering previous work suggesting that individuals with intellectual impairment are more likely to have missing data in ALSPAC (particularly those with more severe ID)(Madley-Dowd et al., 2022), we performed multiple imputation across all association and mediation analyses to mitigate potential bias from missing data (Little & Rubin, 2019). For primary and secondary outcomes, we performed multiple imputations by chained equations, using Stata’s *MI impute* command. One hundred datasets were imputed with 25 burn-in iterations and estimates were combined across imputed datasets using Rubin’s rules, implemented via Stata’s *MI estimate* command. Details on the imputation models applied can be found in Supplementary Note 1. In the case of mediation analyses, we used the inbuilt g-formula imputation commands allowing simultaneous imputation of missing data and mediation analyses, entering in the models the same auxiliary variables we used for the association analyses. In the context of the present study, we present both complete records and imputed data analyses, although we consider as primary the imputed data analyses.

### The role of the funding source

The funders of the study had no role in study design, data analysis, data interpretation, writing of the manuscript, or the decision to submit the manuscript for publication.

## Results

The maximum sample size with data on exposure and at least one outcome measure was 9,407 (49.6% male; 3.6% ID; 0.3% psychotic disorder diagnosis). Full characteristics of our study sample, including covariates and outcome variables are listed in Supplementary Tables 1a (sample with intellectual impairment, IQ < 85) and 1b (sample with intellectual disabilities, IQ < 70). Those with intellectual impairment were more likely to have experienced trauma between ages 5–11. The mothers of those with intellectual impairment were less likely to have a university degree and had a greater prevalence of screening positive for depression. Approximately 65% of the sample had complete data on exposure, psychotic disorder diagnosis (complete for all participants in the eligible sample), and covariates, while 34% of the sample had complete data on exposure, psychotic experiences, and covariates. Participants with complete data were more likely to have a higher socioeconomic background than those with incomplete data (details on the identified patterns can be found in Supplementary Tables 2a & 2b).

### Association analyses

There was some evidence suggesting an association between intellectual impairment and primary care diagnoses of psychotic disorders in crude and adjusted-for covariate models (adjusted OR = 4.84; 95%CI: 1.64, 14.29; Table 1). When considered on the risk difference scale, this odds ratio reflects a small absolute increase in risk among those with intellectual impairment (adjusted marginal risk difference = 1.14%; 95%CI: −0.28%, 2.05%; Table 1); we were unable to report the absolute risk according to the intellectual impairment group due to low counts with a diagnosis among individuals with intellectual impairment. There was also some evidence to support associations between intellectual impairment and psychotic experiences (adjusted OR = 1.59; 95%CI: 0.91, 2.77; adjusted marginal risk difference = 7.69%; 95%CI: −2.58%, 17.96%; Table 1) as well as distressing and/or frequent psychotic experiences (adjusted OR = 1.84; 95%CI: 0.96, 3.54; adjusted marginal risk difference = 7.97%; 95%CI: −2.16%, 19.16%; Table 1) although the confidence intervals of these results crossed the null. Association estimates in complete records analyses were of comparable magnitude, albeit less precise (Supplementary Table 3). In the case of the analyses using intellectual disabilities as the exposure (IQ < 70), there was some evidence of associations, particularly with psychotic disorder, but estimates were highly imprecise due to the small sample size of these analyses (Supplementary Table 4 for analyses using imputed data and Supplementary Table 5 for complete record analyses).

**Table 1.** Associations between intellectual impairment, psychotic disorders, and psychotic experiences in early adulthood from multiple imputation analyses

Outcome	N	Model	OR^1^ (95% CI^2^)	Prevalence of outcome in the sample without II^4^	Prevalence of outcome in the sample with II^4^	Risk difference
Affective & non-affective psychosis diagnosis based on GP records up to age 25	9,407	Unadjusted	3.89 (1.36–11.16)	A^5^	A^5^	0.88 (−0.28–2.05)
		Adjusted for covariates^3^	4.84 (1.64–14.29)	A^5^	A^5^	1.14 (−0.29–2.58)
Psychotic experiences not attributed to sleep or fever up to age 24		Unadjusted	1.64 (0.96–2.80)	17.75 (16.20–19.44)	26.04 (17.74–38.21)	8.66 (−1.93–19.26)
		Adjusted for covariates^3^	1.59 (0.91–2.77)	16.53 (15.08–18.13)	23.70 (15.81–35.52)	7.69 (−2.58–17.96)
Psychotic experiences not attributed to sleep or fever (distressing or frequent) up to age 24		Unadjusted	1.88 (1.00–3.53)	11.83 (10.10–13.86)	20.01 (12.23–32.74)	8.66 (−1.84–19.16)
		Adjusted for covariates^3^	1.84 (0.96–3.54)	10.77 (9.14–12.70)	17.94 (10.76–29.92)	7.97 (−2.16–18.10)

1 Odds ratio.

2 Confidence interval.

3 Adjusted for: child sex (male/female), maternal parity (≤1 child versus ≥2 children), major financial problems in the family when the child was 8 months old (yes/no), maternal highest educational attainment (32 weeks gestation), maternal age (at delivery), maternal Crown-Crisp anxiety scores (18 weeks gestation), maternal depression measured with the Edinburgh Postnatal Depression Scale (EPDS; 18 weeks gestation scores ≥13), car ownership status (owning/not owning, 8 weeks of gestation), maternal marital status (married/separated, 8 weeks of gestation), and home ownership status (owned/rented, 8 weeks of gestation).

4 Intellectual impairment.

5 Exact value omitted to avoid disclosure of cell counts <5 (1.5 = 100 × 5/336, where 336 is the number with ID).

There was little evidence to suggest that individuals with intellectual impairment may be more likely to present with persistent profiles of psychotic experiences. Although the relative risk ratios for the high-frequency persistent profiles were larger than those for the low-frequency persistent and transient profiles, the estimates were highly imprecise (Table 2 for imputed data analyses and Supplementary Table 6 for complete records analyses).

**Table 2.** Associations between ID and longitudinal profiles of psychotic experiences from multiple imputation analyses

Outcome	N	Model	RRR^1^ (95% CI^2^)
No psychotic experiences	9,407	Unadjusted	Ref
Persistent high			3.14 (0.65,15.06)
Persistent low			1.21 (0.45,3.21)
Transient			1.55 (0.85,2.84)
No psychotic experiences		Adjusted for covariates^3^	Ref
Persistent high			2.85 (0.54,15.01)
Persistent low			1.20 (0.44,3.27)
Transient			1.49 (0.80,2.79)

1 Relative risk ratio.

2 Confidence interval.

3 Adjusted for: child sex (male/female), maternal parity (≤1 child versus ≥2 children), major financial problems in the family when the child was 8 months old (yes/no), maternal highest educational attainment (32 weeks gestation), maternal age (at delivery), maternal crown-crisp anxiety scores (18 weeks gestation), maternal depression measured with the Edinburgh Postnatal Depression Scale (EPDS; 18 weeks gestation scores ≥13).

### Mediation analyses

There was some evidence to suggest that childhood trauma may mediate the associations between intellectual impairment and psychotic experiences (effect of exposure on outcome via the mediator, NIE, adjusted OR = 1.09; 95%CI: 1.03–1.15), as well as distressing and/or frequent psychotic experiences (effect of exposure on outcome via the mediator, NIE, adjusted OR = 1.11; 95%CI: 1.03–1.20). Evidence was weaker in the case of psychotic disorders, where traumatic experiences did not appear to mediate the associations with intellectual impairment (Table 3 for imputed data analyses and Supplementary Table 7 for complete records analyses).

**Table 3.** Results of the mediation analyses with childhood traumatic experiences, for the associations between ID, psychotic disorders, and psychotic experiences using imputed data

Outcome	Model	N	TCE^1^; OR^2^ (95%CI^3^)	NDE^4^; OR^2^ (95%CI^3^)	NIE^5^; OR^2^ (95%CI^3^)
Affective & non-affective psychosis diagnosis based on GP records up to age 25	Crude	9,407	3.90 (1.28, 11.90)	3.64 (1.19, 11.11)	1.07 (0.98, 1.17)
	Adjusted^6^		5.82 (1.83, 18.53)	5.53 (1.71, 17.91)	1.03 (0.85, 1.26)
Psychotic experiences not attributed to sleep or fever up to age 24	Crude		0.95 (0.62, 1.46)	0.89 (0.58, 1.36)	1.07 (1.01, 1.13)
	Adjusted^6^		1.07 (0.64, 1.78)	0.98 (0.59, 1.63)	1.09 (1.03, 1.15)
Psychotic experiences not attributed to sleep or fever (distressing or frequent) up to age 24	Crude		0.99 (0.62, 1.59)	0.91 (0.57, 1.45)	1.09 (1.01, 1.17)
	Adjusted^6^		1.19 (0.61, 2.34)	1.07 (0.54, 2.11)	1.11 (1.03, 1.20)

1 Total effect.

2 Odds ratio.

3 Confidence intervals.

4 Natural direct effect.

5 Natural indirect effect.

6 Adjusted for: child sex (male/female), maternal parity (≤1 child versus ≥2 children), major financial problems in the family when the child was 8 months old (yes/no), maternal highest educational attainment (32 weeks gestation), maternal age (at delivery), maternal Crown-Crisp anxiety scores (18 weeks gestation), maternal depression measured with the Edinburgh Postnatal Depression Scale (EPDS; 18 weeks gestation scores ≥13).

## Discussion

Using prospectively collected questionnaires, interviews, and health record linkage data in a population-based birth cohort, we examined the associations between intellectual impairment, psychotic disorders, and psychotic experiences in early adulthood and investigated the factors that may influence them. We found evidence suggesting that intellectual impairment may be associated with psychotic disorders. Although evidence was less consistent in the case of psychotic experiences, traumatic experiences in childhood appeared to mediate their associations with intellectual impairment. The identified relationships were unlikely to be explained by familial, socioeconomic, and demographic factors.

### The relationships of intellectual impairment to psychotic disorders and experiences

Our findings are consistent with a growing body of evidence suggesting that people with intellectual impairment may be at higher risk of psychotic disorders than the general population. The latest and largest study in the field (N = 2,091) found that individuals with intellectual disabilities had a higher risk of psychosis not only compared to the general population but also compared to individuals with other neurodevelopmental conditions (e.g., autism, ADHD)(Strålin & Hetta, 2019). There is an ongoing discussion on the possibility of bias in the existing evidence due to confounding and/or measurement error (measurement error might arise for example, due to the application of diagnostic criteria and tools designed for the general population in individuals with intellectual impairment)(Aman et al., 2016). In our study, we attempted to overcome confounding bias by adjusting our models for several familial, socioeconomic, and demographic factors and found that they are unlikely to explain the identified links (although the possibility of residual confounding cannot be excluded – see Strengths and Limitations section). With regard to measurement error, previous work has indicated that primary care records may underestimate mental health conditions compared to population-based studies such as ALSPAC (Smith et al., 2021). However, in the context of the present study, evidence of associations with psychotic disorder diagnoses was complemented with some evidence of associations between intellectual impairment and subclinical expressions of psychosis liability, and psychotic experiences. Although the evidence was relatively inconsistent across psychotic experience measures, the direction of the association estimates was consistent with the ones identified for psychotic disorders. Moreover, the strongest associations were identified in the case of psychotic experiences that were distressing and/or frequent, a phenotype that is considered to be more strongly related to the subsequent risk of psychotic disorders (Sullivan et al., 2020). Nevertheless, it is important to acknowledge that psychotic experiences were assessed using an s semi-structured tool, PLIKSi, which is not intended for use in individuals with intellectual disabilities, leading therefore to potential under-ascertainment of psychotic experiences in the study sample.

### The mediating role of traumatic experiences in childhood

Our study provides some evidence of the potentially mediating role of traumatic experiences in childhood in the associations between intellectual impairment and psychotic experiences. The evidence was weaker in the case of psychotic disorder and a number of reasons for this could be hypothesized. For example, lack of power may have influenced these analyses, particularly considering that only 0.3% of the total eligible sample (36 individuals) had a diagnosis of psychotic disorder. Moreover, traumatic experiences were measured with a combination of parent and self-reported questionnaires which could lead to underestimation of trauma in the eligible study sample (e.g., parents may underreport the occurrence of traumatic events in the child and/or participants at the age of 24 assessment of traumatic experiences are more likely to drop out).

Although our study is the first to apply a formal counterfactual mediation approach, previous work in a sample of 1,023 adults with intellectual disabilities found that major life events (including but not limited to trauma) were associated with psychiatric conditions in this population (Allan et al., 2007). On this basis, interventions for trauma-related morbidity in this population may substantially improve mental health outcomes. Evidence on the effectiveness of trauma-focused interventions in people with intellectual impairment is promising, indicating that eye movement desensitization and reprocessing (EMDR) as well as trauma-focused cognitive behavioral therapy (CBT) may be effective in this population (McNally et al., 2021), with substantive trials underway (e.g., https://www.isrctn.com/ISRCTN35167485). However, most of the evidence so far comes from case studies and therefore further work is necessary to appraise the appropriateness and effectiveness of these interventions in people with intellectual impairment.

### Strengths and limitations

This is the first study to investigate the links between intellectual impairment, psychotic disorders, and psychotic experiences and assess the possible influence of traumatic experiences in childhood using prospectively collected data from a large population-based cohort. We also used linkage to health and administrative record data, which aided the identification of intellectual impairment and psychotic disorder cases and reduced the impact of attrition and therefore bias due to missing data.

Our study presents several limitations. First, our exposure definition has been broad, including cases of borderline intellectual functioning as well as mild and more severe cases of intellectual disabilities. Despite the fact that analyses using a strict intellectual disabilities definition supported the links with psychosis, the possibility that the associations may differ by the severity of intellectual impairment, cannot be excluded and needs to be investigated further. Second, although we used psychotic experiences as a phenotype reflecting psychotic disorder liability, this might not be the case, as psychotic experiences are associated with several adverse mental health outcomes such as depression and are not specific to psychotic disorders (Legge et al., 2019; Sullivan et al., 2014). Third, our analyses may have been limited by lack of power; this is particularly true for the analyses using the strict intellectual disabilities definition as the exposure. Fourth, although we tried to mitigate the possibility of bias due to missing data using multiple imputations, some bias is still likely to influence the findings of the analyses using psychotic experiences as the outcome (association & and mediation analyses). This is because individuals with intellectual impairment and psychosis are less likely to participate in ALSPAC and the use of psychotic disorder diagnoses as an auxiliary for psychotic experience is unlikely to fully break the link between the outcome and the probability of missing data (Hughes et al., 2019). Fifth, although we adjusted our analyses for a number of potential familial, socioeconomic, and demographic factors, some level of residual confounding is still likely to be present. Sixth, the ALSPAC cohort is not representative of the whole UK population, characterized predominantly by socioeconomically advantaged individuals, and very little ethnic diversity (Boyd et al., 2013). Research using more diverse populations is necessary in order to further elucidate the links between intellectual impairments and psychosis.

## Conclusions

Borderline intellectual functioning and intellectual disabilities are associated with psychotic disorders and experiences into young adulthood. Traumatic experiences in childhood may partially mediate the associations and further research in this area could shape current intervention strategies for psychotic disorders in this population.

## Supporting information

Dardani et al. supplementary material Dardani et al. supplementary material

## Data Availability

Individual-level data from the ALSPAC birth cohort are not publicly available for reasons of clinical confidentiality. Data can be accessed after application to the ALSPAC Executive Team who will respond within 10 working days. Application instructions and data use agreements are available at http://www.bristol.ac.uk/alspac/researchers/access/. The code used to conduct these analyses can be found in the repository: https://github.com/pmadleydowd/BailyThomas-IntellectualDisability-and-MentalHealth

## Supplementary material

The supplementary material for this article can be found at http://doi.org/10.1017/S0033291724003556.
