# Supplementary material for: Psychotic experiences and disorders in adolescents and young adults with borderline intellectual functioning and intellectual disabilities: evidence from a population-based birth cohort in the United Kingdom
Source: Psychol Med. 2025 Feb 5;55:e23. doi: 10.1017/S0033291724003556 (PMC12017352; doi:10.1017/S0033291724003556)
Supplement: Dardani et al. supplementary material [file S0033291724003556sup001.docx]

**Table of Contents**

[**Supplementary Note 1. Information on the multiple imputation analyses.** 2](#_Toc183781442)

[**Supplementary Table 1a. Characteristics of individuals with and without intellectual impairment (IQ<85) in the study sample.** 3](#_Toc183781443)

[**Supplementary Table 1b. Characteristics of individuals with and without intellectual disabilities (IQ<70) in the study sample.** 5](#_Toc183781444)

[**Supplementary Table 2a. Characteristics of the sample with complete data on GP records and covariates.** 7](#_Toc183781445)

[**Supplementary Table 2b. Characteristics of the sample with complete data on psychotic experiences, and covariates.** 9](#_Toc183781446)

[**Supplementary Table 3. Associations between intellectual impairment, psychotic disorders, and psychotic experiences in complete record analyses.** 11](#_Toc183781447)

[**Supplementary Table 4. Associations between intellectual disabilities, psychotic disorders and psychotic experiences in multiple imputation analyses.** 12](#_Toc183781448)

[**Supplementary Table 5. Associations between intellectual disabilities, psychotic disorders and psychotic experiences in complete record analyses.** 13](#_Toc183781449)

[**Supplementary Table 6. Associations between intellectual impairment and the four longitudinal profiles of psychotic experiences in complete record analyses.** 14](#_Toc183781450)

[**Supplementary Table 7. Complete records mediation analyses with childhood trauma for the associations between intellectual impairment, psychotic disorders, and psychotic experiences.** 15](#_Toc183781451)

# **Supplementary Note 1. Information on the multiple imputation analyses.**

In the present analyses we had complete records on intellectual impairment (exposure) and the psychotic disorder diagnoses (outcome). The total number of individuals with complete records on exposure and psychotic disorder diagnoses was 9,407.

On this basis, for the analyses investigating the associations between intellectual impairment and psychotic disorder diagnoses, we imputed the covariates. All covariates were binary variables and therefore were imputed using the logit command in STATA.

In the case of the analyses investigating the associations between ID and psychotic experiences, the total number of individuals with complete records on exposure and outcome was 4,300. We therefore imputed the covariates and the outcome using as an auxiliary variable the psychotic disorder diagnosis. All variables were binary and were imputed using the logit command in STATA. In the case of the analyses investigating the associations between ID and the measure reflecting the persistence and frequency of psychotic experiences (0: “Not present”, 1: “Low-frequency” - experiences occurring less than weekly, 2: “High-frequency” - experiences occurring weekly or daily), we followed the same process for imputation with the exception of using the mlogit command in STATA to impute the nominal outcome variable.

# **Supplementary Table 1a. Characteristics of individuals with and without intellectual impairment (IQ<85) in the study sample.**

|  | **Total Sample** | **Sample without II^1^** | **Sample with II^1^** | **p-value** |
| --- | --- | --- | --- | --- |
| **Variable:** | N=9,407 | N=9,071 | N=336 |  |
| Affective psychosis diagnosis based on GP records | 32 (0.3%) | A^2^ | ≤5 |  |
| sex | | | | 0.085^3^ |
| male | 4,665 (49.6%) | 4,479 (49.4%) | 186 (55.4%) |  |
| female | 4,732 (50.3%) | 4,582 (50.5%) | 150 (44.6%) |  |
| Missing | 10 (0.1%) | 10 (0.1%) | 0 (0.0%) |  |
| parity | | | | 0.062^3^ |
| <=1 | 6,511 (69.2%) | 6,290 (69.3%) | 221 (65.8%) |  |
| >=2 | 1,677 (17.8%) | 1,601 (17.6%) | 76 (22.6%) |  |
| Missing | 1,219 (13.0%) | 1,180 (13.0%) | 39 (11.6%) |  |
| Maternal education | | | | <0.001^3^ |
| Not University graduate | 6,988 (74.3%) | 6,705 (73.9%) | 283 (84.2%) |  |
| University graduate | 940 (10.0%) | 931 (10.3%) | 9 (2.7%) |  |
| Missing | 1,479 (15.7%) | 1,435 (15.8%) | 44 (13.1%) |  |
| Maternal age at delivery, mean (SD) | 28.0 (4.9) | 28.0 (5.0) | 28.0 (4.9) | 0.88^4^ |
| Major financial problems | | | | 0.68^3^ |
| No | 6,280 (66.8%) | 6,055 (66.8%) | 225 (67.0%) |  |
| Yes | 1,063 (11.3%) | 1,021 (11.3%) | 42 (12.5%) |  |
| Missing | 2,064 (21.9%) | 1,995 (22.0%) | 69 (20.5%) |  |
| Maternal anxiety during pregnancy, mean (SD) | 4.9 (3.5) | 4.9 (3.5) | 5.2 (3.6) | 0.11^4^ |
| Maternal depression during pregnancy, EPDS≥12 | | | | <0.001^3^ |
| Not present | 6,305 (67.0%) | 6,098 (67.2%) | 207 (61.6%) |  |
| Present | 1,334 (14.2%) | 1,259 (13.9%) | 75 (22.3%) |  |
| Missing | 1,768 (18.8%) | 1,714 (18.9%) | 54 (16.1%) |  |
| Missing | 5,107 (54.3%) | 4,937 (54.4%) | 170 (50.6%) |  |
| Psychotic experiences until 18 and/or 24 not attributed to sleep or fever | | | | 0.29^3^ |
| Not present | 3,779 (40.2%) | 3,636 (40.1%) | 143 (42.6%) |  |
| Present | 521 (5.5%) | 498 (5.5%) | 23 (6.8%) |  |
| Missing | 5,107 (54.3%) | 4,937 (54.4%) | 170 (50.6%) |  |
| Psychotic experiences until 18 and/or 24 not attributed to sleep or fever, distressing or frequent | | | | 0.22^3^ |
| Not present | 4,020 (42.7%) | 3,868 (42.6%) | 152 (45.2%) |  |
| Present | 280 (3.0%) | 266 (2.9%) | 14 (4.2%) |  |
| Missing | 5,107 (54.3%) | 4,937 (54.4%) | 170 (50.6%) |  |
| Trajectory of psychotic experiences | | | | 0.29^3^ |
| No psychotic experiences | 1,555 (16.5%) | A^2^ | A^2^ |  |
| Persistent High | 22 (0.2%) | A^2^ | ≤5 |  |
| Persistent Low | 135 (1.4%) | A^2^ | ≤5 |  |
| Transient | 414 (4.4%) | A^2^ | A^2^ |  |
| Missing | 7,281 (77.4%) | A^2^ | A^2^ |  |
| Trajectory of psychotic experiences | | | | 0.34^3^ |
| No psychotic experiences | 1,555 (16.5%) | 1,509 (16.6%) | 46 (13.7%) |  |
| Persistent (High and Low) | 157 (1.7%) | 150 (1.7%) | 7 (2.1%) |  |
| Transient | 414 (4.4%) | 395 (4.4%) | 19 (5.7%) |  |
| Missing | 7,281 (77.4%) | 7,017 (77.4%) | 264 (78.6%) |  |
| Home ownership status | | | | 0.056^3^ |
| Owned | 6,201 (65.9%) | 5,991 (66.0%) | 210 (62.5%) |  |
| Rented | 2,034 (21.6%) | 1,944 (21.4%) | 90 (26.8%) |  |
| Missing | 1,172 (12.5%) | 1,136 (12.5%) | 36 (10.7%) |  |
| Maternal marital status | | | | 0.74^3^ |
| Married | 6,264 (66.6%) | 6,039 (66.6%) | 225 (67.0%) |  |
| Separated | 2,015 (21.4%) | 1,940 (21.4%) | 75 (22.3%) |  |
| Missing | 1,128 (12.0%) | 1,092 (12.0%) | 36 (10.7%) |  |
| Traumatic experiences between ages 5-11 | | | | <0.001^3^ |
| Not present | 5,044 (53.6%) | 4,872 (53.7%) | 172 (51.2%) |  |
| Present | 2,475 (26.3%) | 2,357 (26.0%) | 118 (35.1%) |  |
| Missing | 1,888 (20.1%) | 1,842 (20.3%) | 46 (13.7%) |  |
| Car ownership status | | | | 0.003^3^ |
| Owning | 7,400 (78.7%) | 7,150 (78.8%) | 250 (74.4%) |  |
| Not owning | 848 (9.0%) | 800 (8.8%) | 48 (14.3%) |  |
| Missing | 1,159 (12.3%) | 1,121 (12.4%) | 38 (11.3%) |  |
| Autism diagnosis based on GP records | | | | <0.001^3^ |
| Not present | 9,352 (99.4%) | 9,035 (99.6%) | 317 (94.3%) |  |
| Present | 55 (0.6%) | 36 (0.4%) | 19 (5.7%) |  |
| ADHD diagnosis based on GP records | | | | <0.001^3^ |
| Not present | 9,270 (98.5%) | 8,949 (98.7%) | 321 (95.5%) |  |
| Present | 137 (1.5%) | 122 (1.3%) | 15 (4.5%) |  |
| 1: Intellectual Impairment, broad definition, IQ<85  2: the value cannot be presented to avoid secondary disclosure.  3: Pearson x^2^ test  4: Independent samples t-test. | | | | |

# **Supplementary Table 1b. Characteristics of individuals with and without intellectual disabilities (IQ<70) in the study sample.**

|  | **Total Sample** | **Sample without ID^1^** | **Sample with ID^1^** | **p-value** |
| --- | --- | --- | --- | --- |
| **Variable:** | N=9,407 | N=9,287 | N=120 |  |
| Affective psychosis diagnosis based on GP records | 32 (0.3%) | A^2^ | ≤5 |  |
| sex | | | | 0.028^3^ |
| male | 4,665 (49.6%) | 4,591 (49.4%) | 74 (61.7%) |  |
| female | 4,732 (50.3%) | 4,686 (50.5%) | 46 (38.3%) |  |
| Missing | 10 (0.1%) | 10 (0.1%) | 0 (0.0%) |  |
| parity | | | | <0.001^3^ |
| <=1 | 6,511 (69.2%) | 6,445 (69.4%) | 66 (55.0%) |  |
| >=2 | 1,677 (17.8%) | 1,641 (17.7%) | 36 (30.0%) |  |
| Missing | 1,219 (13.0%) | 1,201 (12.9%) | 18 (15.0%) |  |
| Maternal education | | | | 0.26^3^ |
| Not University graduate | 6,988 (74.3%) | 6,897 (74.3%) | 91 (75.8%) |  |
| University graduate | 940 (10.0%) | 933 (10.0%) | 7 (5.8%) |  |
| Missing | 1,479 (15.7%) | 1,457 (15.7%) | 22 (18.3%) |  |
| Maternal age at delivery, mean (SD) | 28.0 (4.9) | 28.0 (4.9) | 28.8 (5.3) | 0.082^4^ |
| Major financial problems | | | | 0.033^3^ |
| Not present | 6,280 (66.8%) | 6,211 (66.9%) | 69 (57.5%) |  |
| Present | 1,063 (11.3%) | 1,050 (11.3%) | 13 (10.8%) |  |
| Missing | 2,064 (21.9%) | 2,026 (21.8%) | 38 (31.7%) |  |
| Maternal anxiety during pregnancy, mean (SD) | 4.9 (3.5) | 4.9 (3.5) | 5.5 (3.8) | 0.11^4^ |
| Maternal depression during pregnancy, EPDS≥12 | | | | <0.001^3^ |
| Not present | 6,305 (67.0%) | 6,241 (67.2%) | 64 (53.3%) |  |
| Present | 1,334 (14.2%) | 1,304 (14.0%) | 30 (25.0%) |  |
| Missing | 1,768 (18.8%) | 1,742 (18.8%) | 26 (21.7%) |  |
| Psychotic experiences until 18 and/or 24 not attributed to sleep or fever | | | | <0.001^3^ |
| Not present | 3,779 (40.2%) | A^2^ | ≤5 |  |
| Present | 521 (5.5%) | A^2^ | ≤5 |  |
| Missing | 5,107 (54.3%) | A^2^ | ≤5 |  |
| Psychotic experiences unitl 18 and/or 24 not attributed to sleep or fever, distressing or frequent | | | | <0.001^3^ |
| Not present | 4,020 (42.7%) | A^2^ | ≤5 |  |
| Present | 280 (3.0%) | A^2^ | ≤5 |  |
| Missing | 5,107 (54.3%) | A^2^ | ≤5 |  |
| Trajectory of psychotic experiences | | | | 0.002^3^ |
| No psychotic experiences | 1,555 (16.5%) | A^2^ | ≤5 |  |
| Persistent High | 22 (0.2%) | A^2^ | ≤5 |  |
| Persistent Low | 135 (1.4%) | A^2^ | ≤5 |  |
| Transient | 414 (4.4%) | A^2^ | ≤5 |  |
| Missing | 7,281 (77.4%) | A^2^ | ≤5 |  |
| Trajectory of psychotic experiences | | | | <0.001^3^ |
| No psychotic experiences | 1,555 (16.5%) | A^2^ | ≤5 |  |
| Persistent Overall | 157 (1.7%) | A^2^ | ≤5 |  |
| Transient | 414 (4.4%) | A^2^ | ≤5 |  |
| Missing | 7,281 (77.4%) | A^2^ | ≤5 |  |
| Home ownership status | | | | 0.004^3^ |
| Owned | 6,201 (65.9%) | 6,139 (66.1%) | 62 (51.7%) |  |
| Rented | 2,034 (21.6%) | 1,996 (21.5%) | 38 (31.7%) |  |
| Missing | 1,172 (12.5%) | 1,152 (12.4%) | 20 (16.7%) |  |
| Maternal marital status | | | | 0.26^3^ |
| Married | 6,264 (66.6%) | 6,190 (66.7%) | 74 (61.7%) |  |
| Separated | 2,015 (21.4%) | 1,989 (21.4%) | 26 (21.7%) |  |
| Missing | 1,128 (12.0%) | 1,108 (11.9%) | 20 (16.7%) |  |
| Traumatic experiences between ages 5-11 | | | | 0.12^3^ |
| Not present | 5,044 (53.6%) | 4,986 (53.7%) | 58 (48.3%) |  |
| Present | 2,475 (26.3%) | 2,446 (26.3%) | 29 (24.2%) |  |
| Missing | 1,888 (20.1%) | 1,855 (20.0%) | 33 (27.5%) |  |
| Car ownership status | | | | <0.001^3^ |
| Owning | 7,400 (78.7%) | 7,327 (78.9%) | 73 (60.8%) |  |
| Not owning | 848 (9.0%) | 822 (8.9%) | 26 (21.7%) |  |
| Missing | 1,159 (12.3%) | 1,138 (12.3%) | 21 (17.5%) |  |
| Autism diagnosis based on GP records | | | | <0.001^3^ |
| Not present | 9,352 (99.4%) | 9,250 (99.6%) | 102 (85.0%) |  |
| Present | 55 (0.6%) | 37 (0.4%) | 18 (15.0%) |  |
| ADHD diagnosis based on GP records | | | | <0.001^3^ |
| Not present | 9,270 (98.5%) | 9,160 (98.6%) | 110 (91.7%) |  |
| Present | 137 (1.5%) | 127 (1.4%) | 10 (8.3%) |  |
| 1: Intellectual Disabilities, strict definition, IQ<70  2: the value cannot be presented to avoid secondary disclosure.  3: Pearson x^2^ test  4: Independent samples t-test. | | | | |

# **Supplementary Table 2a. Characteristics of the sample with complete data on GP records and covariates.**

|  | **Total Sample** | **Sample with complete data** | **Sample with incomplete data^1^** | **p-value** |
| --- | --- | --- | --- | --- |
|  | N=9,407 | N=6,105 | N=3,302 |  |
| Intellectual impairment^2^ (broad definition, IQ<85) | | | | 0.25^3^ |
| Not present | 9,071 (96.4%) | 5,877 (96.3%) | 3,194 (96.7%) |  |
| Present | 336 (3.6%) | 228 (3.7%) | 108 (3.3%) |  |
| Intellectual disabilties^2^ (strict definition, IQ<70) | | | | 0.022^3^ |
| Not present | 9,287 (98.7%) | 6,039 (98.9%) | 3,248 (98.4%) |  |
| Present | 120 (1.3%) | 66 (1.1%) | 54 (1.6%) |  |
| Affective psychosis diagnosis based on GP records | | | | 0.012^3^ |
| Not present | 9,375 (99.7%) | 6,091 (99.8%) | 3,284 (99.5%) |  |
| Present | 32 (0.3%) | 14 (0.2%) | 18 (0.5%) |  |
| sex | | | | <0.001^3^ |
| male | 4,665 (49.6%) | 3,055 (50.0%) | 1,610 (48.8%) |  |
| female | 4,732 (50.3%) | 3,050 (50.0%) | 1,682 (50.9%) |  |
| Missing | 10 (0.1%) | 0 (0.0%) | 10 (0.3%) |  |
| parity | | | | <0.001^3^ |
| <=1 | 6,511 (69.2%) | 4,923 (80.6%) | 1,588 (48.1%) |  |
| >=2 | 1,677 (17.8%) | 1,182 (19.4%) | 495 (15.0%) |  |
| Missing | 1,219 (13.0%) | 0 (0.0%) | 1,219 (36.9%) |  |
| Maternal education | | | | <0.001^3^ |
| Not University graduate | 6,988 (74.3%) | 5,314 (87.0%) | 1,674 (50.7%) |  |
| University graduate | 940 (10.0%) | 791 (13.0%) | 149 (4.5%) |  |
| Missing | 1,479 (15.7%) | 0 (0.0%) | 1,479 (44.8%) |  |
| Maternal age at delivery, mean (SD) | 28.0 (4.9) | 28.7 (4.6) | 26.5 (5.3) | <0.001^4^ |
| Major financial problems | | | | <0.001^3^ |
| Not present | 6,280 (66.8%) | 5,253 (86.0%) | 1,027 (31.1%) |  |
| Present | 1,063 (11.3%) | 852 (14.0%) | 211 (6.4%) |  |
| Missing | 2,064 (21.9%) | 0 (0.0%) | 2,064 (62.5%) |  |
| Maternal anxiety during pregnancy, mean (SD) | 4.9 (3.5) | 4.7 (3.5) | 5.6 (3.7) | <0.001^4^ |
| Maternal depression during pregnancy, EPDS≥12 | | | | <0.001^3^ |
| Not present | 6,305 (67.0%) | 5,154 (84.4%) | 1,151 (34.9%) |  |
| Present | 1,334 (14.2%) | 951 (15.6%) | 383 (11.6%) |  |
| Missing | 1,768 (18.8%) | 0 (0.0%) | 1,768 (53.5%) |  |
| Psychotic experiences until 18 and/or 24 not attributed to sleep or fever | | | | <0.001^3^ |
| Not present | 3,779 (40.2%) | 2,848 (46.7%) | 931 (28.2%) |  |
| Present | 521 (5.5%) | 367 (6.0%) | 154 (4.7%) |  |
| Missing | 5,107 (54.3%) | 2,890 (47.3%) | 2,217 (67.1%) |  |
| Psychotic experiences unitl 18 and/or 24 not attributed to sleep or fever, distressing or frequent | | | | <0.001^3^ |
| Not present | 4,020 (42.7%) | 3,020 (49.5%) | 1,000 (30.3%) |  |
| Present | 280 (3.0%) | 195 (3.2%) | 85 (2.6%) |  |
| Missing | 5,107 (54.3%) | 2,890 (47.3%) | 2,217 (67.1%) |  |
| Trajectory of psychotic experiences | | | | <0.001^3^ |
| No psychotic experiences | 1,555 (16.5%) | 1,249 (20.5%) | 306 (9.3%) |  |
| Persistent High | 22 (0.2%) | 13 (0.2%) | 9 (0.3%) |  |
| Persistent Low | 135 (1.4%) | 107 (1.8%) | 28 (0.8%) |  |
| Transient | 414 (4.4%) | 324 (5.3%) | 90 (2.7%) |  |
| Missing | 7,281 (77.4%) | 4,412 (72.3%) | 2,869 (86.9%) |  |
| Trajectory of psychotic experiences | | | | <0.001^3^ |
| No psychotic experiences | 1,555 (16.5%) | 1,249 (20.5%) | 306 (9.3%) |  |
| Persistent Overall | 157 (1.7%) | 120 (2.0%) | 37 (1.1%) |  |
| Transient | 414 (4.4%) | 324 (5.3%) | 90 (2.7%) |  |
| Missing | 7,281 (77.4%) | 4,412 (72.3%) | 2,869 (86.9%) |  |
| Home ownership status | | | | <0.001^3^ |
| Owned | 6,201 (65.9%) | 4,949 (81.1%) | 1,252 (37.9%) |  |
| Rented | 2,034 (21.6%) | 1,156 (18.9%) | 878 (26.6%) |  |
| Missing | 1,172 (12.5%) | 0 (0.0%) | 1,172 (35.5%) |  |
| Maternal marital status | | | | <0.001^3^ |
| Married | 6,264 (66.6%) | 4,895 (80.2%) | 1,369 (41.5%) |  |
| Separated | 2,015 (21.4%) | 1,210 (19.8%) | 805 (24.4%) |  |
| Missing | 1,128 (12.0%) | 0 (0.0%) | 1,128 (34.2%) |  |
| Traumatic experiences between ages 5-11 | | | | <0.001^3^ |
| Not present | 5,044 (53.6%) | 3,846 (63.0%) | 1,198 (36.3%) |  |
| Present | 2,475 (26.3%) | 1,922 (31.5%) | 553 (16.7%) |  |
| Missing | 1,888 (20.1%) | 337 (5.5%) | 1,551 (47.0%) |  |
| Car ownership status | | | | <0.001^3^ |
| Owning | 7,400 (78.7%) | 5,677 (93.0%) | 1,723 (52.2%) |  |
| Not owning | 848 (9.0%) | 428 (7.0%) | 420 (12.7%) |  |
| Missing | 1,159 (12.3%) | 0 (0.0%) | 1,159 (35.1%) |  |
| Autism diagnosis based on GP records | | | | 0.13^3^ |
| Not present | 9,352 (99.4%) | 6,064 (99.3%) | 3,288 (99.6%) |  |
| Present | 55 (0.6%) | 41 (0.7%) | 14 (0.4%) |  |
| ADHD diagnosis based on GP records | | | | <0.001^3^ |
| Not present | 9,270 (98.5%) | 6,039 (98.9%) | 3,231 (97.8%) |  |
| Present | 137 (1.5%) | 66 (1.1%) | 71 (2.2%) |  |
| 1: Missing data in covariate or more  2: Intellectual impairment was the primary exposure across all analyses  3: Pearson x2 test  4: Independent samples t-test. | | | | |

**Supplementary Table 2b. Characteristics of the sample with complete data on psychotic experiences, and covariates.**

|  | **Total Sample** | **Sample with complete data** | **Sample with incomplete data^1^** | **p-value** |
| --- | --- | --- | --- | --- |
|  | N=9,407 | N=3,215 | N=6,192 |  |
| Intellectual impairment^2^ (broad definition, IQ<85) | | | | 0.12^3^ |
| Not present | 9,071 (96.4%) | 3,087 (96.0%) | 5,984 (96.6%) |  |
| Present | 336 (3.6%) | 128 (4.0%) | 208 (3.4%) |  |
| Intellectual disabiliteis^2^ (strict definition, IQ<70) | | | | 0.002^3^ |
| Not present | 9,287 (98.7%) | 3,190 (99.2%) | 6,097 (98.5%) |  |
| Present | 120 (1.3%) | 25 (0.8%) | 95 (1.5%) |  |
| Affective psychosis diagnosis based on GP records | | | | 0.47^3^ |
| Not present | 9,375 (99.7%) | 3,206 (99.7%) | 6,169 (99.6%) |  |
| Present | 32 (0.3%) | 9 (0.3%) | 23 (0.4%) |  |
| sex | | | | <0.001^3^ |
| male | 4,665 (49.6%) | 1,396 (43.4%) | 3,269 (52.8%) |  |
| female | 4,732 (50.3%) | 1,819 (56.6%) | 2,913 (47.0%) |  |
| Missing | 10 (0.1%) | 0 (0.0%) | 10 (0.2%) |  |
| parity | | | | <0.001^3^ |
| <=1 | 6,511 (69.2%) | 2,684 (83.5%) | 3,827 (61.8%) |  |
| >=2 | 1,677 (17.8%) | 531 (16.5%) | 1,146 (18.5%) |  |
| Missing | 1,219 (13.0%) | 0 (0.0%) | 1,219 (19.7%) |  |
| Maternal education | | | | <0.001^3^ |
| Not University graduate | 6,988 (74.3%) | 2,625 (81.6%) | 4,363 (70.5%) |  |
| University graduate | 940 (10.0%) | 590 (18.4%) | 350 (5.7%) |  |
| Missing | 1,479 (15.7%) | 0 (0.0%) | 1,479 (23.9%) |  |
| Maternal age at delivery, mean (SD) | 28.0 (4.9) | 29.4 (4.5) | 27.2 (5.0) | <0.001^4^ |
| Major financial problems | | | | <0.001^3^ |
| Not present | 6,280 (66.8%) | 2,806 (87.3%) | 3,474 (56.1%) |  |
| Present | 1,063 (11.3%) | 409 (12.7%) | 654 (10.6%) |  |
| Missing | 2,064 (21.9%) | 0 (0.0%) | 2,064 (33.3%) |  |
| Maternal anxiety during pregnancy, mean (SD) | 4.9 (3.5) | 4.5 (3.4) | 5.2 (3.6) | <0.001^4^ |
| Maternal depression during pregnancy, EPDS≥12 | | | | <0.001^3^ |
| Not present | 6,305 (67.0%) | 2,784 (86.6%) | 3,521 (56.9%) |  |
| Present | 1,334 (14.2%) | 431 (13.4%) | 903 (14.6%) |  |
| Missing | 1,768 (18.8%) | 0 (0.0%) | 1,768 (28.6%) |  |
| Psychotic experiences until 18 and/or 24 not attributed to sleep or fever | | | | <0.001^3^ |
| Not present | 3,779 (40.2%) | 2,848 (88.6%) | 931 (15.0%) |  |
| Present | 521 (5.5%) | 367 (11.4%) | 154 (2.5%) |  |
| Missing | 5,107 (54.3%) | 0 (0.0%) | 5,107 (82.5%) |  |
| Psychotic experiences unitl 18 and/or 24 not attributed to sleep or fever, distressing or frequent | | | | <0.001^3^ |
| Not present | 4,020 (42.7%) | 3,020 (93.9%) | 1,000 (16.1%) |  |
| Present | 280 (3.0%) | 195 (6.1%) | 85 (1.4%) |  |
| Missing | 5,107 (54.3%) | 0 (0.0%) | 5,107 (82.5%) |  |
| Trajectory of psychotic experiences | | | | <0.001^3^ |
| No psychotic experiences | 1,555 (16.5%) | 1,249 (38.8%) | 306 (4.9%) |  |
| Persistent High | 22 (0.2%) | 13 (0.4%) | 9 (0.1%) |  |
| Persistent Low | 135 (1.4%) | 107 (3.3%) | 28 (0.5%) |  |
| Transient | 414 (4.4%) | 324 (10.1%) | 90 (1.5%) |  |
| Missing | 7,281 (77.4%) | 1,522 (47.3%) | 5,759 (93.0%) |  |
| Trajectory of psychotic experiences | | | | <0.001^3^ |
| No psychotic experiences | 1,555 (16.5%) | 1,249 (38.8%) | 306 (4.9%) |  |
| Persistent Overall | 157 (1.7%) | 120 (3.7%) | 37 (0.6%) |  |
| Transient | 414 (4.4%) | 324 (10.1%) | 90 (1.5%) |  |
| Missing | 7,281 (77.4%) | 1,522 (47.3%) | 5,759 (93.0%) |  |
| Home ownership status | | | | <0.001^3^ |
| Owned | 6,201 (65.9%) | 2,797 (87.0%) | 3,404 (55.0%) |  |
| Rented | 2,034 (21.6%) | 418 (13.0%) | 1,616 (26.1%) |  |
| Missing | 1,172 (12.5%) | 0 (0.0%) | 1,172 (18.9%) |  |
| Maternal marital status | | | | <0.001^3^ |
| Married | 6,264 (66.6%) | 2,671 (83.1%) | 3,593 (58.0%) |  |
| Separated | 2,015 (21.4%) | 544 (16.9%) | 1,471 (23.8%) |  |
| Missing | 1,128 (12.0%) | 0 (0.0%) | 1,128 (18.2%) |  |
| Traumatic experiences between ages 5-11 | | | | <0.001^3^ |
| Not present | 5,044 (53.6%) | 1,994 (62.0%) | 3,050 (49.3%) |  |
| Present | 2,475 (26.3%) | 1,193 (37.1%) | 1,282 (20.7%) |  |
| Missing | 1,888 (20.1%) | 28 (0.9%) | 1,860 (30.0%) |  |
| Car ownership status | | | | <0.001^3^ |
| Owning | 7,400 (78.7%) | 3,078 (95.7%) | 4,322 (69.8%) |  |
| Not owning | 848 (9.0%) | 137 (4.3%) | 711 (11.5%) |  |
| Missing | 1,159 (12.3%) | 0 (0.0%) | 1,159 (18.7%) |  |
| Autism diagnosis based on GP records | | | | 0.73^3^ |
| Not present | 9,352 (99.4%) | 3,195 (99.4%) | 6,157 (99.4%) |  |
| Present | 55 (0.6%) | 20 (0.6%) | 35 (0.6%) |  |
| ADHD diagnosis based on GP records | | | | <0.001^3^ |
| Not present | 9,270 (98.5%) | 3,193 (99.3%) | 6,077 (98.1%) |  |
| Present | 137 (1.5%) | 22 (0.7%) | 115 (1.9%) |  |
| 1: Missing data in covariate or more  2: Intellectual impairment was the primary exposure across all analyses  3: Pearson x2 test  4: Independent samples t-test. | | | | |

# **Supplementary Table 3. Associations between intellectual impairment, psychotic disorders, and psychotic experiences in complete record analyses.**

| **outcome** | **model** | **Total Sample** | **Sample with outcome** | **Sample with exposure** | **Sample with exposure and outcome** | **OR^1^ (95% Cis^2^)** | **Prevalence outcome in sample without II^3^** | **Prevalence outcome in sample with II^3^** | **RD^4^ (95% CIs)** |
| --- | --- | --- | --- | --- | --- | --- | --- | --- | --- |
| Affective psychosis diagnosis based on GP records | Unadjusted | 9407 | A^5^ | A^5^ | ≤5 | 3.89 (1.36 - 11.16) | 0.31 (0.21 , 0.45) | 1.19 (0.45 , 3.15) | 0.88 (-0.28 , 2.05) |
| Affective psychosis diagnosis based on GP records | Crude | 6105 | A^5^ | A^5^ | ≤5 | 7.11 (1.97 - 25.66) | 0.19 (0.10 , 0.34) | 1.32 (0.43 , 4.05) | 1.13 (-0.35 , 2.61) |
| Affective psychosis diagnosis based on GP records | Adjusted | 6105 | A^5^ | A^5^ | ≤5 | 9.20 (2.41 - 35.17) | 0.13 (0.06 , 0.28) | 1.20 (0.37 , 3.89) | 1.47 (-0.44 , 3.38) |
| Psychotic experiences until 18 and/or 24 not attributed to sleep or fever | Unadjusted | 4300 | 521 | 166 | 23 | 1.17 (0.75 - 1.84) | 12.05 (11.09 , 13.08) | 13.86 (9.48 , 20.25) | 1.81 (-3.54 , 7.16) |
| Psychotic experiences until 18 and/or 24 not attributed to sleep or fever | Crude | 3215 | 367 | 128 | 17 | 1.20 (0.71 - 2.02) | 11.34 (10.27 , 12.51) | 13.28 (8.53 , 20.68) | 1.94 (-4.04 , 7.93) |
| Psychotic experiences until 18 and/or 24 not attributed to sleep or fever | Adjusted | 3215 | 367 | 128 | 17 | 1.11 (0.65 - 1.90) | 10.80 (9.74 , 11.97) | 11.88 (7.50 , 18.80) | 1.12 (-4.61 , 6.84) |
| Psychotic experiences until 18 and/or 24 not attributed to sleep or fever, distressing or frequent | Unadjusted | 4300 | 280 | 166 | 14 | 1.34 (0.76 - 2.35) | 6.43 (5.73 , 7.23) | 8.43 (5.11 , 13.92) | 2.00 (-2.29 , 6.29) |
| Psychotic experiences until 18 and/or 24 not attributed to sleep or fever, distressing or frequent | Crude | 3215 | 195 | 128 | 9 | 1.18 (0.59 - 2.36) | 6.03 (5.24 , 6.93) | 7.03 (3.75 , 13.20) | 1.01 (-3.50 , 5.51) |
| Psychotic experiences until 18 and/or 24 not attributed to sleep or fever, distressing or frequent | Adjusted | 3215 | 195 | 128 | 9 | 1.13 (0.56 - 2.28) | 5.72 (4.94 , 6.62) | 6.40 (3.35 , 12.19) | 0.71 (-3.67 , 5.08) |
| 1: Odds ratio  2: Confidence interval  3: Intellectual impairment  4: Risk difference  5: The value cannot be presented to avoid secondary disclosure | | | | | | | | | |

# **Supplementary Table 4. Associations between intellectual disabilities, psychotic disorders and psychotic experiences in multiple imputation analyses.**

| **outcome** | **model** | **OR^1^ (95% Cis^2^)** | **FMI^3^ (exp)** | **FMI^3^ (large)** | **Prevalence outcome in sample without ID^4^** | **Prevalence outcome in sample with ID^4^** | **RD^5^ (95%CIs)** |
| --- | --- | --- | --- | --- | --- | --- | --- |
| Affective psychosis diagnosis based on GP records | Unadjusted | 5.23 (1.24-22.14) | 0 | 0 | 0.32 (0.23 - 0.46) | 1.67 (0.42 - 6.59) | 1.34 (-0.95 - 3.64) |
| Affective psychosis diagnosis based on GP records | Adjusted | 6.77 (1.53-30.00) | 0.0134973 | 0.4447662 | 0.23 (0.14 - 0.37) | 1.50 (0.36 - 6.23) | 1.79 (-1.14 - 4.71) |
| Psychotic experiences until 18 and/or 24 not attributed to sleep or fever | Unadjusted | 10.55 (3.33-33.44) | 0.8753515 | 0.8753515 | 17.77 (16.02 - 19.72) | 67.34 (42.70 - 106.21) | 50.93 (25.47 - 76.40) |
| Psychotic experiences until 18 and/or 24 not attributed to sleep or fever | Adjusted | 10.79 (3.22-36.19) | 0.8801902 | 0.8801902 | 16.54 (14.91 - 18.35) | 64.80 (39.10 - 107.38) | 49.22 (23.26 - 75.19) |
| Psychotic experiences until 18 and/or 24 not attributed to sleep or fever, distressing or frequent | Unadjusted | 9.81 (1.55-62.21) | 0.9500183 | 0.9500183 | 11.92 (9.92 - 14.33) | 52.23 (17.55 - 155.41) | 45.46 (7.99 - 82.93) |
| Psychotic experiences until 18 and/or 24 not attributed to sleep or fever, distressing or frequent | Adjusted | 10.63 (1.50-75.33) | 0.9524471 | 0.9524471 | 10.89 (9.03 - 13.12) | 50.35 (15.65 - 161.92) | 44.94 (7.37 - 82.50) |
| 1: Odds Ratio  2: Confidence Interval  3: Fraction of missing information (multiple imputation measure)  4: Intellectual Disabilities  5: Risk difference | | | | | | | |

# **Supplementary Table 5. Associations between intellectual disabilities, psychotic disorders and psychotic experiences in complete record analyses.**

| **outcome** | **model** | **Total Sample** | **Sample with outcome** | **Sample with exposure** | **Sample with exposure and outcome** | **OR^1^ (95% Cis^2^)** | **Prevalence outcome in sample without ID^3^** | **Prevalence outcome in sample with ID^3^** | **RD (95% CIs^4^)** |
| --- | --- | --- | --- | --- | --- | --- | --- | --- | --- |
| Affective psychosis diagnosis based on GP records | Unadjusted | 9407 | A^5^ | A^5^ | ≤5 | 5.23 (1.24 - 22.14) | 0.32 (0.23 , 0.46) | 1.67 (0.42 , 6.59) | 1.34 (-0.95 , 3.64) |
| Affective psychosis diagnosis based on GP records | Crude_complete_exposure_outcome_covariates | 6105 | A^5^ | A^5^ | ≤5 | 15.70 (3.44 - 71.55) | 0.20 (0.11 , 0.35) | 3.03 (0.77 , 11.86) | 2.83 (-1.31 , 6.97) |
| Affective psychosis diagnosis based on GP records | Adjusted_complete_exposure_outcome_covariates | 6105 | A^5^ | A^5^ | ≤5 | 17.79 (3.68 - 86.13) | 0.15 (0.07 , 0.29) | 2.52 (0.60 , 10.57) | 3.12 (-1.45 , 7.69) |
| Psychotic experiences until 18 and/or 24 not attributed to sleep or fever | Unadjusted | 4300 | A^5^ | A^5^ | ≤5 | 1.12 (0.39 - 3.21) | 12.11 (11.17 , 13.13) | 13.33 (5.35 , 33.20) | 1.23 (-10.98 , 13.43) |
| Psychotic experiences until 18 and/or 24 not attributed to sleep or fever | Crude_complete_exposure_outcome_covariates | 3215 | A^5^ | A^5^ | ≤5 | 1.48 (0.51 - 4.35) | 11.38 (10.33 , 12.54) | 16.00 (6.52 , 39.28) | 4.62 (-9.79 , 19.03) |
| Psychotic experiences until 18 and/or 24 not attributed to sleep or fever | Adjusted_complete_exposure_outcome_covariates | 3215 | A^5^ | A^5^ | ≤5 | 1.43 (0.48 - 4.27) | 10.81 (9.76 , 11.97) | 14.71 (5.85 , 37.02) | 4.02 (-9.93 , 17.98) |
| Psychotic experiences until 18 and/or 24 not attributed to sleep or fever, distressing or frequent | Unadjusted | 4300 | A^5^ | A^5^ | ≤5 | 1.60 (0.48 - 5.31) | 6.49 (5.79 , 7.27) | 10.00 (3.42 , 29.26) | 3.51 (-7.25 , 14.27) |
| Psychotic experiences until 18 and/or 24 not attributed to sleep or fever, distressing or frequent | Crude_complete_exposure_outcome_covariates | 3215 | A^5^ | A^5^ | ≤5 | 2.13 (0.63 - 7.18) | 6.02 (5.25 , 6.90) | 12.00 (4.15 , 34.69) | 5.98 (-6.78 , 18.75) |
| Psychotic experiences until 18 and/or 24 not attributed to sleep or fever, distressing or frequent | Adjusted_complete_exposure_outcome_covariates | 3215 | A^5^ | A^5^ | ≤5 | 2.21 (0.65 - 7.56) | 5.70 (4.93 , 6.58) | 11.75 (4.03 , 34.27) | 6.29 (-6.71 , 19.28) |
| 1: Odds ratio  2: Confidence interval  3: Intellectual disabilities  4: Risk difference  5: The value cannot be presented to avoid secondary disclosure | | | | | | | | | |

# **Supplementary Table 6. Associations between intellectual impairment and the four longitudinal profiles of psychotic experiences in complete record analyses.**

| **Trajectory** | **model** | **N** | **RRR^1^ (95% CIs^2^)** |
| --- | --- | --- | --- |
| No psychotic experiences | Unadjusted^3^ | 2126 | Ref |
| Persistent High |  | 2126 | 3.28 (0.74, 14.45) |
| Persistent Low |  | 2126 | 1.26 (0.49, 3.23) |
| Transient |  | 2126 | 1.58 (0.91, 2.72) |
| No psychotic experiences | Unadjusted^4^ | 1714 | Ref |
| Persistent High |  | 1714 | 2.93 (0.37, 23.17) |
| Persistent Low |  | 1714 | 0.67 (0.16, 2.82) |
| Transient |  | 1714 | 1.45 (0.76, 2.78) |
| No psychotic experiences | Adjusted^5^ | 1714 | Ref |
| Persistent High |  | 1714 | 2.32 (0.28, 19.18) |
| Persistent Low |  | 1714 | 0.69 (0.16, 2.95) |
| Transient |  | 1714 | 1.39 (0.72, 2.68) |
| 1: Relative Risk Ratio  2: Confidence Interval  3: Sample with complete records on exposure, outcome  4: Sample with complete records on exposure, outcome, covariates.  5: Adjusted for: child sex (male/female), maternal parity (≤1 child versus ≥ 2 children), major financial problems in the family when the child was 8 months old (yes/no), maternal highest educational attainment (32 weeks gestation), maternal age (at delivery), maternal Crown-Crisp anxiety scores (18 weeks gestation), maternal depression measured with the Edinburgh Postnatal Depression Scale (EPDS; 18 weeks gestation scores ≥ 13). | | | |

# **Supplementary Table 7. Complete records mediation analyses with childhood trauma for the associations between intellectual impairment, psychotic disorders, and psychotic experiences.**

| **outcome** | **Model** | **N** | **TCE^1^; OR^2^ (95%CIs^3^)** | **NDE^4^; OR^2^ (95%CIs^3^)** | **NIE^5^; OR^2^ (95%CIs^3^)** |
| --- | --- | --- | --- | --- | --- |
| Affective psychosis diagnosis based on GP records | Unadjusted^6^ | 7519 | 5.95 (1.83, 19.28) | 5.44 (1.69, 17.47) | 1.09 (0.99, 1.20) |
| Affective psychosis diagnosis based on GP records | Unadjusted^7^ | 5890 | 6.79 (1.83, 25.25) | 6.11 (1.64, 22.75) | 1.11 (0.96, 1.28) |
| Affective psychosis diagnosis based on GP records | Adjusted^8^ | 5890 | 8.97 (2.35, 34.29) | 8.20 (2.17, 30.91) | 1.09 (0.96, 1.25) |
| Psychotic experiences until 18 and/or 24 not attributed to sleep or fever | Unadjusted^6^ | 7519 | 1.09 (0.68, 1.75) | 1.06 (0.67, 1.69) | 1.03 (0.98, 1.08) |
| Psychotic experiences until 18 and/or 24 not attributed to sleep or fever | Unadjusted^7^ | 5890 | 1.19 (0.72, 1.97) | 1.16 (0.71, 1.90) | 1.03 (0.98, 1.09) |
| Psychotic experiences until 18 and/or 24 not attributed to sleep or fever | Adjusted^8^ | 5890 | 1.16 (0.71, 1.89) | 1.14 (0.70, 1.85) | 1.02 (0.98, 1.07) |
| Psychotic experiences until 18 and/or 24 not attributed to sleep or fever, distressing or frequent | Unadjusted^6^ | 7519 | 1.30 (0.70, 2.41) | 1.26 (0.68, 2.32) | 1.03 (0.97, 1.09) |
| Psychotic experiences until 18 and/or 24 not attributed to sleep or fever, distressing or frequent | Unadjusted^7^ | 5890 | 1.26 (0.61, 2.60) | 1.21 (0.59, 2.49) | 1.04 (0.97, 1.11) |
| Psychotic experiences until 18 and/or 24 not attributed to sleep or fever, distressing or frequent | Adjusted^8^ | 5890 | 1.24 (0.60, 2.55) | 1.21 (0.59, 2.47) | 1.03 (0.97, 1.09) |
| 1: Total effect  2: Odds Ratio  3: Confidence Intervals  4: Natural Direct Effect  5: Natural Indirect Effect  6: Sample with complete records on exposure, mediator, outcome  7: Sample with complete records on exposure, mediator, outcome, covariates.  8: Adjusted for: child sex (male/female), maternal parity (≤1 child versus ≥ 2 children), major financial problems in the family when the child was 8 months old (yes/no), maternal highest educational attainment (32 weeks gestation), maternal age (at delivery), maternal Crown-Crisp anxiety scores (18 weeks gestation), maternal depression measured with the Edinburgh Postnatal Depression Scale (EPDS; 18 weeks gestation scores ≥ 13). | | | | | |
